# Ultrastructural analyses in the hippocampus CA1 field in *Shank3*-deficient mice

**DOI:** 10.1186/s13229-015-0036-x

**Published:** 2015-06-30

**Authors:** Neha Uppal, Rishi Puri, Frank Yuk, William G M Janssen, Ozlem Bozdagi-Gunal, Hala Harony-Nicolas, Dara L Dickstein, Joseph D Buxbaum, Patrick R Hof

**Affiliations:** Fishberg Department of Neuroscience, Icahn School of Medicine at Mount Sinai, One Gustave L Levy Place, Box 1639, New York, NY 10029 USA; Friedman Brain Institute, Icahn School of Medicine at Mount Sinai, New York, NY USA; Seaver Autism Center for Research and Treatment, Icahn School of Medicine at Mount Sinai, New York, NY USA; Graduate School of Biomedical Sciences, Icahn School of Medicine at Mount Sinai, New York, NY USA; Department of Psychiatry, Icahn School of Medicine at Mount Sinai, New York, NY USA; The Sheryl and Daniel R. Tishman Cognitive Neurophysiology Laboratory, Department of Pediatrics, Albert Einstein College of Medicine, 1225 Morris Park Avenue, Bronx, NY 10461 USA

**Keywords:** Autism, Shank3, Electron microscopy, CA1, Hippocampus, Stratum radiatum, Synapse

## Abstract

**Background:**

The genetics of autism spectrum disorder (hereafter referred to as “autism”) are rapidly unfolding, with a significant increase in the identification of genes implicated in the disorder. Many of these genes are part of a complex landscape of genetic variants that are thought to act together to cause the behavioral phenotype associated with autism. One of the few single-locus causes of autism involves a mutation in the SH3 and multiple ankyrin repeat domains 3 (*SHANK3*) gene. Previous electrophysiological studies in mice with *Shank3* mutations demonstrated impairment in synaptic long-term potentiation, suggesting a potential disruption at the synapse.

**Methods:**

To understand how variants in *SHANK3* would lead to such impairments and manifest in the brain of patients with autism, we assessed the presence of synaptic pathology in *Shank3-*deficient mice at 5 weeks and 3 months of age, focusing on the stratum radiatum of the CA1 field. This study analyzed both *Shank3* heterozygous and homozygous mice using an electron microscopy approach to determine whether there is a morphological correlate to the synaptic functional impairment.

**Results:**

As both synaptic strength and plasticity are affected in *Shank3-*deficient mice, we hypothesized that there would be a reduction in synapse density, postsynaptic density length, and perforated synapse density. No differences were found in most parameters assessed. However, *Shank3* heterozygotes had significantly higher numbers of perforated synapses at 5 weeks compared to 3 months of age and significantly higher numbers of perforated synapses compared to 5-week-old wildtype and *Shank3* homozygous mice.

**Conclusions:**

Although this finding represents preliminary evidence for ultrastructural alterations, it suggests that while major structural changes seem to be compensated for in *Shank3-*deficient mice, more subtle morphological alterations, affecting synaptic structure, may take place in an age-dependent manner.

## Background

The behavioral symptoms that currently define autism have yet to be rooted in a cause, though it is generally understood that these symptoms originate from alterations in the brain. Many studies have validated this hypothesis, as regionally specific abnormalities including reduced neuronal size, decreased neuronal number, and cortical disorganization are present in individuals with autism (e.g., [1–6]). The abnormalities in neuronal architecture imply corresponding disruptions in communication among neurons, which likely manifest at the synapse. Of particular relevance is the growing number of synaptic function-related genes implicated in autism. The Src homology 3 and multiple ankyrin repeat domains 3 (*SHANK3*) gene, which encodes for the postsynaptic scaffolding protein SHANK3/ProSAP2, has been identified as one of the few genes which can by itself lead to autism when mutated. *SHANK3* was identified as the cause of the neurobehavioral manifestations of the 22q13 deletion syndrome, or the Phelan-McDermid syndrome (PMS; [7]). Patients with PMS not only exhibit developmental, intellectual, and language delays as well as motor deficits but also frequently demonstrate autistic behaviors [8–11]. Although patients with 22q13 deletions have varying deletion sizes, *SHANK3* is a gene that has been consistently implicated [12] and was later found to be exclusively affected through point mutations in some individuals with PMS [10, 13–15], leading to the conclusion that this gene is likely a strong candidate gene for autism as well. Upon further analysis, *SHANK3* variants were identified in patients ascertained with autism [9, 11, 13, 16–18]. Given its crucial role in the etiology of PMS and autism, studying the impact of a loss of *SHANK3* will allow for a detailed assessment of cellular and molecular mechanisms that underlie pathology.

SHANK3 belongs to a family of SHANK proteins, all of which have multiple domains for protein-protein interaction [19, 20]. Each of these domains interacts with specific proteins, in particular with cytoskeletal proteins, cell adhesion proteins, and ionotropic and metabotropic glutamate receptors [21–25], some of which are also implicated in autism (e.g., NLGN3, NLGN4, SHANK1, SHANK2). SHANK3 is strongly expressed at the postsynaptic density (PSD), as it is part of a molecular assembly (which includes PSD-95/GKAP/Shank/Homer) which comprises approximately 27 % of the total protein molecules in the PSD; Shank3 is estimated to comprise ~5 % of the total protein molecules in the PSD [26]. To determine the effect of a loss of one copy of *SHANK3*, as observed in patients with PMS [11], Bozdagi and colleagues [27] created a mouse with a disruption in full-length *Shank3*, which is a form known to be specifically disrupted in patients with autism (e.g., [18]). Functionally, the authors demonstrated that *Shank3* heterozygotes display significant impairments in long-term potentiation (LTP) maintenance when recording from the stratum radiatum in the CA1. Furthermore, they reported a significant decrease in α-amino-3-hydroxy-5-methyl-4-isoxazole propionic acid (AMPA) receptor-mediated field potentials in the *Shank3* heterozygotes as well as a reduction in the amount of glutamate receptor subunit 1 (GluA1), an AMPA receptor subunit. Subsequently, Yang and colleagues [28] corroborated the electrophysiologic impairments in *Shank3* heterozygous mice and found impairments in both LTP induction and maintenance in *Shank3* homozygous mice*.* While Shank3 protein is widely expressed throughout the brain, it is concentrated in the thalamus, cerebellum, and hippocampus [29]. Because of the impairment in LTP seen in *Shank3* heterozygous and homozygous mice, Shank3 expression in the hippocampus was particularly interesting to assess.

Given these findings and the crucial role of *SHANK3* in the etiology of PMS and autism, this study examined ultrastructural alterations in the above *Shank3* mouse model of autism. The primary morphological parameters assessed in the current study were synapse density, perforated synapse density, and PSD length in the *Shank3-*deficient mice, due to the likelihood these parameters would be affected by a loss of Shank3, given its role in the PSD and LTP (e.g., [27, 30]). Differences in PSD length would indicate a change in the ratio of plastic and stable spines, as PSD length is correlated with spine head size and type. We also assessed perforated synapses, which are distinguished by a discontinuous PSD despite being part of a single synapse. Perforated synapses have been implicated in many functions in the context of learning and memory, including maintenance of spatial learning ability, activity-dependent synaptic plasticity, and LTP induction [31–35]. The formation of a perforation in the synapse, which transiently occurs during LTP induction, is thought to enhance the efficacy of synaptic transmission due to the increase in PSD area and therefore increase in AMPA- and *N*-methyl-D-aspartic acid (NMDA)-receptor content [33, 34, 36]. Their formation, however, remains a subject of debate, with some postulating that non-perforated synapses become perforated, as a transient intermediary structure, to then form two separate synapses (for example, [37]), others postulating that perforated and non-perforated synapses are completely separate populations of synapses that develop in unique ways (for example, [38]), and others suggesting that perforations transiently develop in non-perforated synapses to briefly increase the efficacy of the synapse, which will later form back into its original non-perforated structure (for example, [39]). Despite the debate on perforated synapse origin and development, their role in increasing the efficacy of a synapse is generally agreed upon and is the reason we are assessing their presence in our mouse model. Changes in synapse functioning, as measured through morphological changes, would likely lead to alterations in normal neuronal functioning. Alterations in neuronal functioning, in turn, might be reflected in the behavioral changes that currently characterize individuals with disorders such as PMS and autism. There are very few studies that have assessed hippocampal differences in postmortem brains from this patient population and that have identified neuronal abnormalities that may affect synaptic functioning. Neuronal abnormalities in the hippocampus in patients with autism include reduction in neuron volume, dendritic branching, and nucleus volume [3, 6], although to our knowledge there are no studies that have quantified changes at the synaptic level in patients with autism. Unfortunately, as of yet, there have been no postmortem studies in individuals with PMS nor specifically on individuals with autism that have a disruption in *SHANK3*; however, we can presume that this region is likely also affected in these individuals, as they present with similar behavioral symptoms.

In the current study, synaptic measures were assessed at two time points: 5 weeks, corresponding to an early adolescence stage near sexual maturity when the relevant brain region is undergoing constant synaptic pruning, and 3 months, corresponding to a young adult that is socially mature and has a reduced rate of synaptic turnover. These ages were consistent with previous studies conducted on this mouse model [27, 28], which allowed for direct comparison of the synaptic measures in this study with electrophysiological and behavioral measures from earlier studies. Overall, this study aimed to clarify the effect of *Shank3* deficiency on synapse morphology.

## Methods

### Animals

All procedures were compliant with the National Institute of Health Guidelines for the Care and Use of Experimental Animals and were approved by the Institutional Animal Care and Use Committee at the Icahn School of Medicine at Mount Sinai. *Shank3* heterozygous and homozygous mice were generated using homologous recombination to delete the ankyrin repeat domain of *Shank3*. This constitutive knockout mouse model of *Shank3* disruption has been previously characterized in detail [27, 28]. The study included 15 C57BL/6 male mice (5 weeks old: 5 *Shank3* homozygous mice, 5 *Shank3* heterozygous mice, and 5 wildtype mice) and 15 C57BL/6 male mice (3 months old: 5 *Shank3* homozygous mice, 5 *Shank3* heterozygous mice, and 5 wildtype mice). Mice were group-housed and were given standard mouse chow and water *ad libitum*.

### Perfusion and tissue processing

Prior to perfusion, mice were anesthetized with 15 % chloral hydrate. The mice were perfused with 1 % cold paraformaldehyde (pH 7.2–7.4) in phosphate buffer saline (PBS) for 1.5 min. This was followed by a solution of 4 % paraformaldehyde in 0.1 M PBS and 0.125 % glutaraldehyde for 10.5 min at 5 ml/min. The brain was then removed from the skull and postfixed in a 4 % paraformaldehyde/0.125 % glutaraldehyde solution overnight at 4 °C. The brain was washed in phosphate buffer (PB), cut into 250-μm-thick sections using a vibratome, and stored in PB overnight. The sections were cryoprotected the next day in a graded glycerol/PB solution. After remaining in a 30 % glycerol solution overnight, the CA1 was manually microdissected into blocks (four per animal) that underwent cryosubstitution and low-temperature embedding as described in [40].

Once the polymerization was complete, the hardened capsules were removed from the cryosubstitution system and stored at room temperature. Block faces were trimmed to include the stratum pyramidale, stratum radiatum, stratum lacunosum-moleculare, and white matter. At least eight consecutive ultrathin sections were cut at 90 nm with a diamond knife (Diatome, Bienne, Switzerland) on an ultramicrotome (Reichert-Jung) and mounted onto a Formvar-supported slot grid (Electron Microscopy Sciences).

### Morphological analysis

Serial section imaging was carried out at 75 kV on a Hitachi H-7000 transmission electron microscope (Hitachi High Technologies America) with an AMT Advantage CCD camera (Advanced Microscopy Techniques). Eight sets of five serial sections of distinct regions in the stratum radiatum of the CA1 were imaged at ×15,000. Electron micrographs were adjusted for brightness, contrast, and sharpness using Adobe Photoshop (Photoshop CS4, version 11.0.2) prior to being analyzed. All morphological analysis was carried out in Adobe Photoshop.

**Fig. 1:**
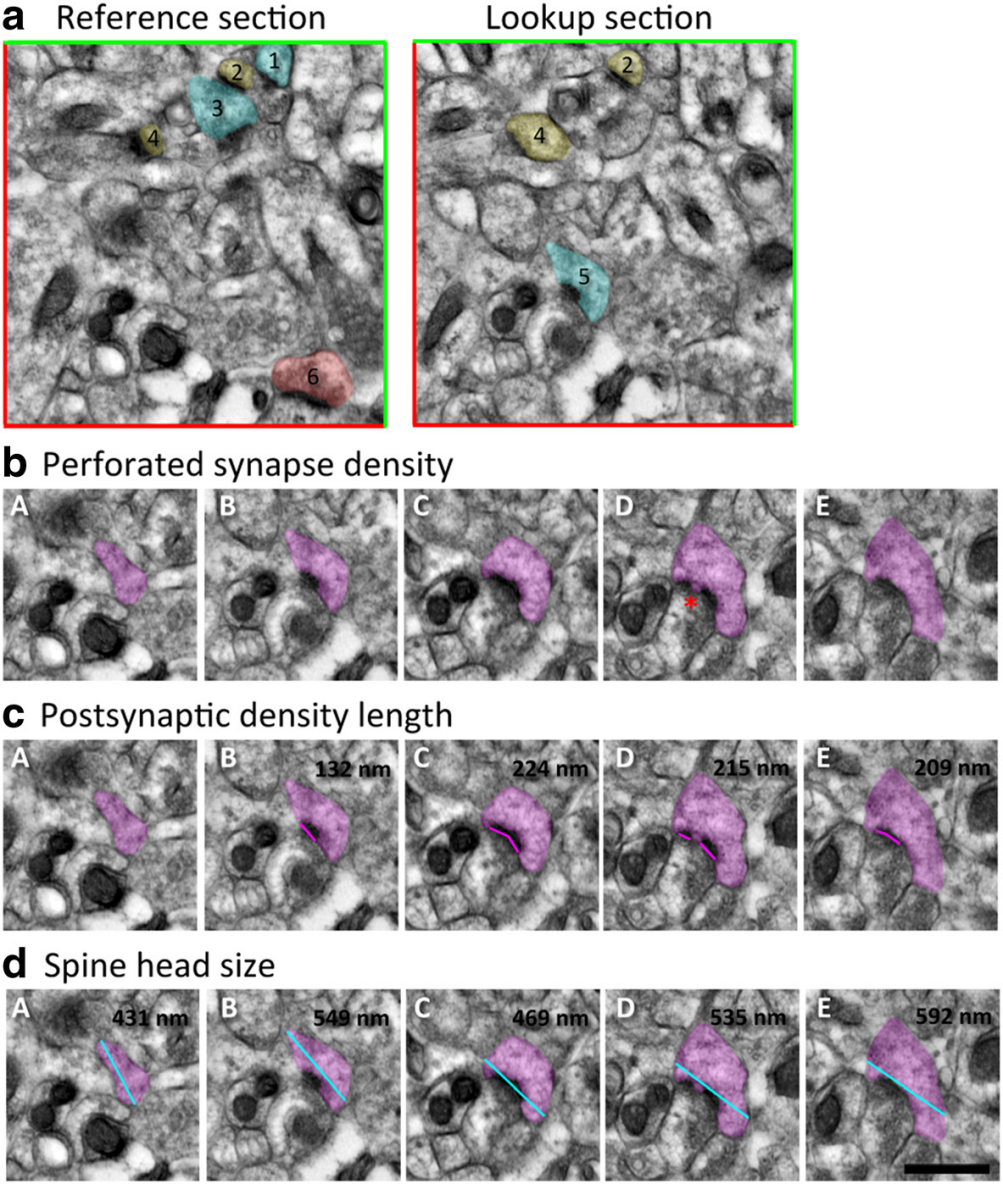
The disector method. **a** Representative picture of a reference and lookup section in a set of serial images labeled *A–E*, with *A* as reference and *B* as lookup; note the inclusion (*green*) and exclusion (*red*) lines bordering the boundaries of images. Synapses that are present in both the reference and lookup sections are not counted towards synapse density (synapses *2* and *4*; *yellow*) nor are synapses in which the pre- or postsynapse touch the exclusion lines (synapse *6*; *red*). Each unique synapse in the lookup section (synapse *5*; *blue*) is followed through the rest of the series to identify perforated synapses (*red asterisk*; **b**) and measure postsynaptic density length, indicated with *pink lines* (**c**), and spine head size, indicated by *blue lines* (**d**). Scale bar = 500 nm

Synapse density and perforated synapse density were analyzed using the disector method (Fig. 1; [41]). Asymmetric axospinous synapses were defined as having an asymmetric PSD, presynaptic vesicles, a clear synaptic cleft, and an evident spine head. All synapses present in the first (“reference”) section, but not the second (“lookup”), were used for calculations of synapse density, along with each synapse that was present in the second section, but not the first. To calculate perforated synapse density, all unique synapses from the second section were followed through the remaining three sections to determine whether the synapses were perforated (defined as a clear break in the PSD). PSD length was measured in each section with a resolution of 0.5 μm/113 pixels. The longest measured PSD length was used as the representative measure for that synapse. This process was also completed for spine head size, measuring the widest point of the spine head parallel to the PSD. The longest length of spine head was used as the representative measure of that synapse. This entire process was then repeated for each set but using the last two sections as the reference and lookup sections; this allowed for approximately 400–600 synapses recorded per animal. The disector area was 58.65 μm^2^, and disector height was 180 nm; therefore, the synapse density was calculated as the total number of counted synapses from both the first and second sections divided by the total volume of the disector (10.56 μm^3^).

### Statistical analysis

We compared two ages and three genotypes of mice. Both the 5-week and 3-month group had an *n* = 5 per genotype, with a total of 15 mice each. Comparisons of potential genotypic variations within one age group were determined using one-way analysis of variance (ANOVA). To reduce type I error while comparing several means, a Tukey *post hoc* test was used to calculate whether there were significant differences between pairs of genotypes. Calculations were performed with GraphPad Prism (version 5). To determine whether there was an interaction between age and genotype on the parameters assessed, we used two-way ANOVA to assess for significant differences and corrected for multiple comparisons using the Tukey *post hoc* test. Calculations were performed with SPSS (version 20). A *p* value of 0.05 was used as the criterion for statistical significance.

## Results

**Fig. 2:**
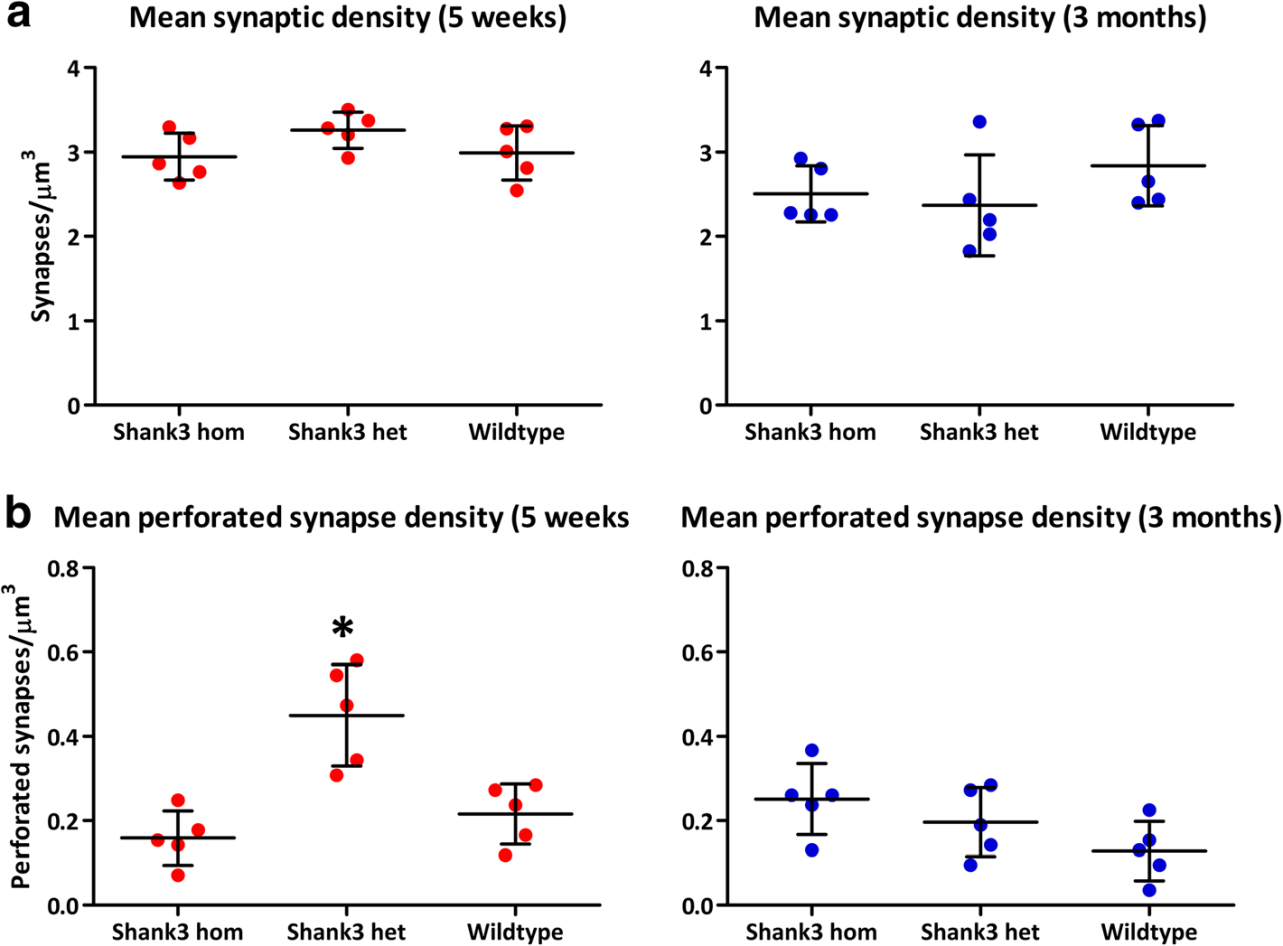
Synapse density and perforated synapse density across ages and genotypes. No change in density of synapses (**a**) was found in 5-week-old mice (*left*) and 3-month-old mice (*right*). A significant increase in perforated synapses (**b**) is shown in 5-week-old *Shank3* heterozygous mice (*left*), but no difference was seen at 3 months (*right*). Standard deviation indicated with *vertical black lines*, mean indicated by *horizontal black line*; *denotes significance

**Fig. 3:**
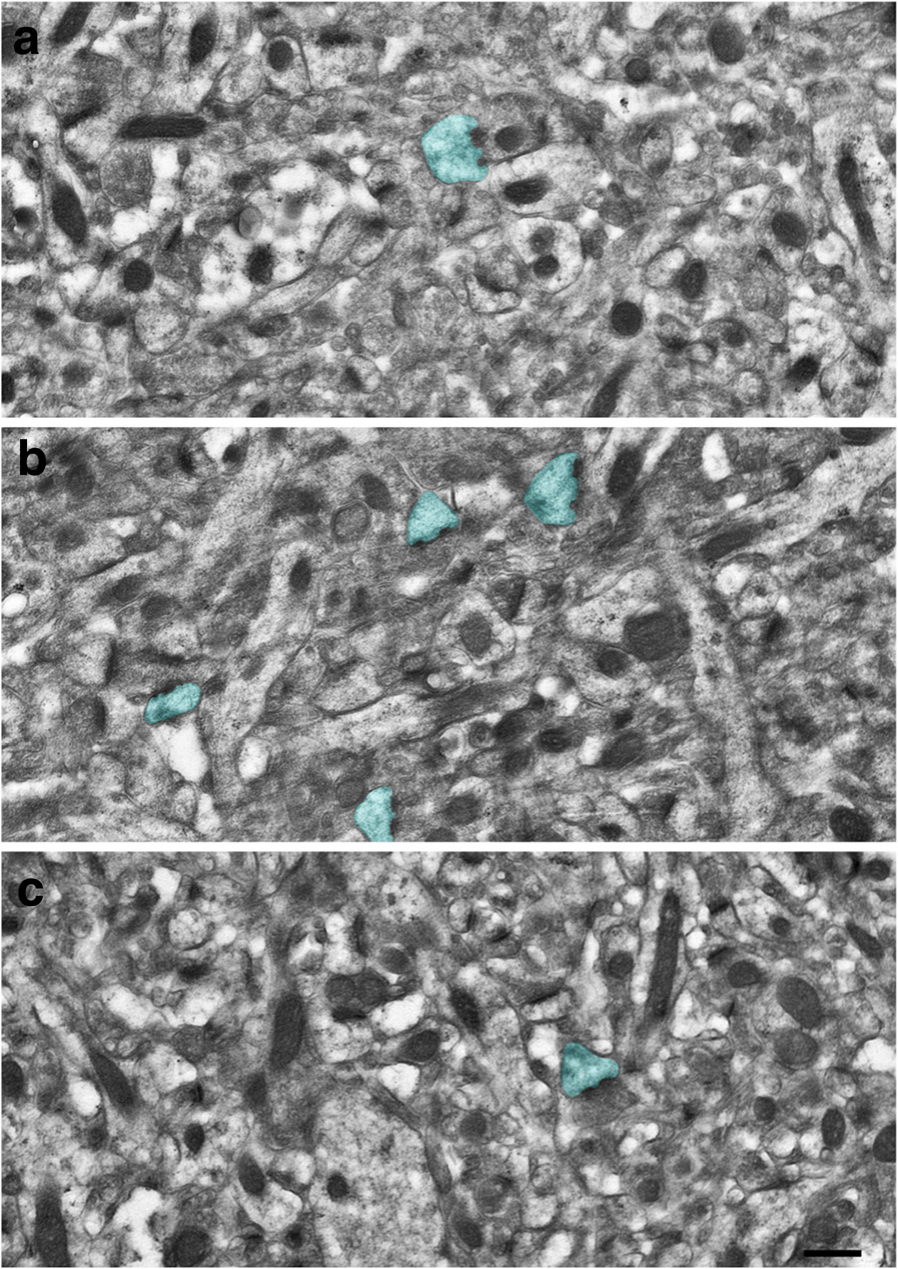
Increased perforated synapses in *Shank3* heterozygous mice at 5 weeks old. Representative photomicrograph of a serial section in a *Shank3* homozygote (**a**) *Shank3* heterozygote (**b**), and wildtype (**c**). Note the increased number of perforated synapses, shaded in *blue*, present in (**b**). Scale bar = 500 nm

The first measure of synaptic pathology we assessed in these mice was overall synaptic density and perforated synapse density. No difference was found in synapse density between *Shank3* heterozygous, *Shank3* homozygous, and wildtype 5-week-old mice (*F*_(2,12)_ = 1.915, *p* = 0.19; Fig. 2a). This result remained consistent into adulthood, with 3-month-old mice also showing no difference in synapse density across the three genotypes (*F*_(2,12)_ = 1.256, *p* = 0.32; Fig. 2a), though 5-week-old mice had significantly greater synapse density than 3-month-old mice (*p* = 0.002). There was no significant interaction between genotype and age for synapse density (*F*_(2,24)_ = 2.254, *p* = 0.127). However, perforated synapse density was significantly different in 5-week-old mice across genotypes (*F*_(2,12)_ = 15.09, *p* = 0.0005), with significantly higher perforated synapse density in *Shank3* heterozygous mice as compared to wildtype and *Shank3* homozygous mice (*p* < 0.05; Figs. 2b and 3). This increase did not persist into adulthood, as 3-month-old mice did not have significant differences across genotypes in perforated synapse density (*F*_(2,12)_ = 3.047, *p* = 0.085; Fig. 2b). A two-way ANOVA revealed a significant interaction between age and genotype in perforated synapse density (*F*_(2,24)_ = 10.569, *p* = 0.001). To follow up on this interaction, we carried out a one-way ANOVA controlling for age, with genotype as a factor. As expected, there was a significant difference in perforated synapse density between genotypes (F_(2, 479)_ = 17.85, *p* < 0.001). A Tukey *post hoc* test revealed that *Shank3* heterozygotes had significantly greater perforated synapse density than wildtype (*p* < 0.001) and *Shank3* homozygotes (*p* < 0.001), though the difference was not significant between wildtype and *Shank3* homozygotes (*p* = 0.848).

**Fig. 4:**
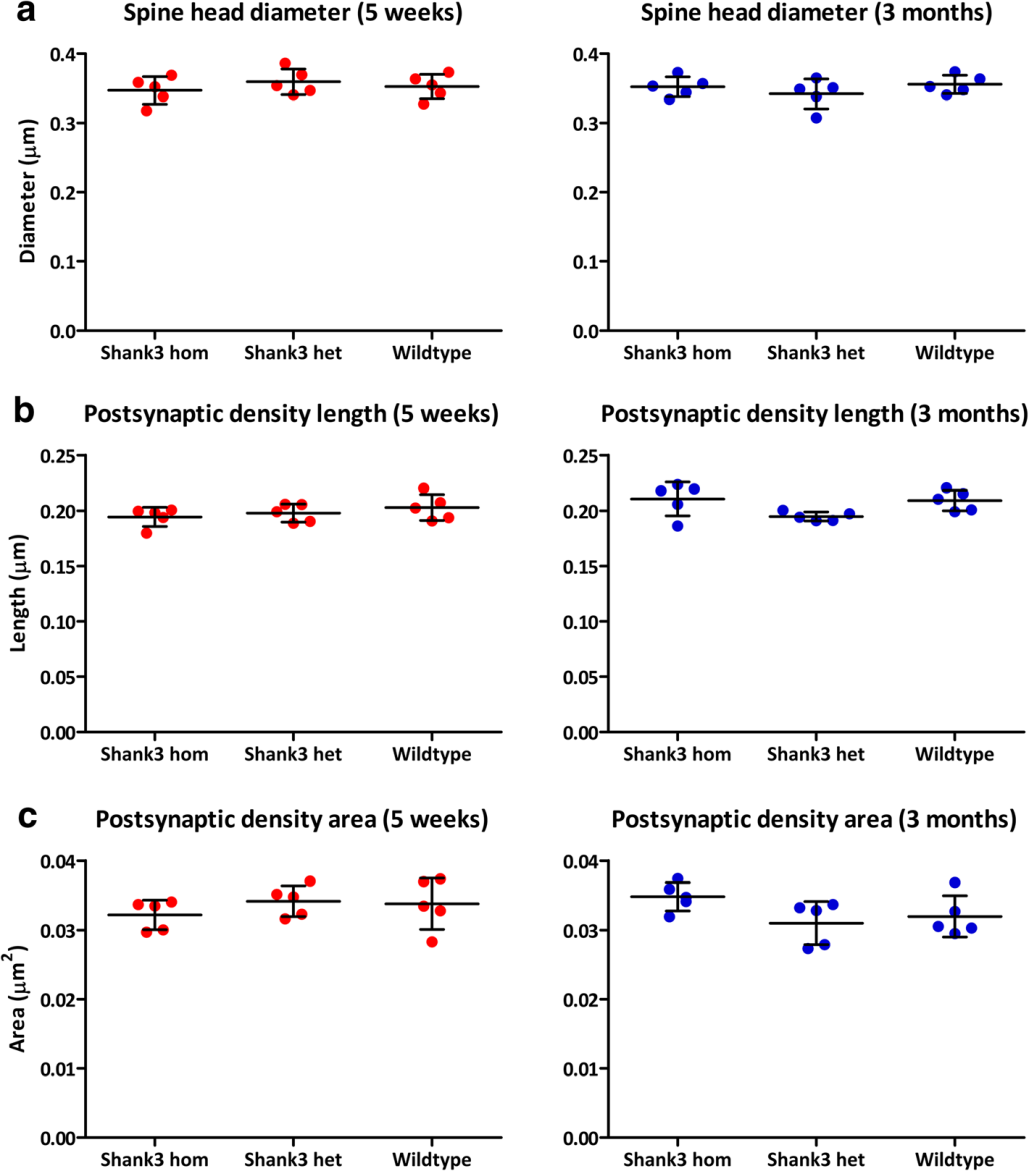
No change in morphologic measures across ages and genotypes. No change was found in spine head size (**a**), postsynaptic density length (**b**), or postsynaptic density area (**c**) in 5-week-old mice (*left column*) and in 3-month-old mice (*right column*). Standard deviation indicated with *vertical black lines*, mean indicated by *horizontal black line*

We then analyzed 5-week-old and 3-month-old mice for changes in spine head size, length of the PSD, and area of the PSD. There was no difference in average spine head size in the 5-week-old mice (*F*_(2,12)_ = 0.551, *p* = 0.59; Fig. 4a) or 3-month-old mice (*F*_(2,12)_ = 0.899, *p* = 0.433, Fig. 4a) nor was there an interaction effect of age and genotype on spine head size (*F*_(2,24)_ = 1.243, *p* = 0.306). There was no significant difference between genotypes in the PSD length in 5-week-old mice (*F*_(2,12)_ = 0.976, *p* = 0.405; Fig. 4b), 3-month-old mice (*F*_(2,12)_ = 3.477, *p* = 0.064, Fig. 4b), or across development (*F*_(2,24)_ = 2.308, *p* = 0.121). PSD area was also the same across genotypes in 5-week-old mice (*F*_(2,12)_ = 0.714, *p* = 0.509, Fig. 4c), 3-month-old mice (*F*_(2,12)_ = 2.067, *p* = 0.115, Fig. 4c), and across both developmental stages (*F*_(2,24)_ = 3.021, *p* = 0.068).

## Discussion

The Shank family of postsynaptic scaffolding proteins consists of three members (Shank1, Shank2, and Shank3; [21, 22]). Shank1 and Shank3 mRNA transcripts are strongly expressed in the pyramidal and molecular layers of the hippocampus, indicating dendritic localization, whereas Shank2 mRNA transcripts are restricted to cell bodies [29, 42]. Shank2 protein localizes at the PSD early (prior to PSD-95 and NR1; [30]) and is thought to act as an initial “organizer” of the PSD; Shank3 is recruited next, interacting with Homer1b to create a complex on which postsynaptic proteins, such as GKAP, can bind [43]; lastly, Shank1 enters after the PSD-95/GKAP complex is in place to stabilize synapses and may have a role in activity-induced regulation of spine head size [22, 44, 45].

With the loss of a major synaptic scaffolding protein, we expected to find significant morphologic changes in the *Shank3-*deficient mice. However, *Shank3* heterozygous and homozygous mice exhibited no difference in spine head size, PSD length, and PSD area when compared to wildtype mice. This result suggests that the absence of Shank3 may be compensated for or may lead to deficits that are only observed early in development. Two obvious candidates for this compensation are Shank1 and Shank2. Shank2 is more similar to Shank3 in its recruitment to the synapse and its protein structure, making it a more likely candidate for compensation [20]. In fact, Shank3 was thought to compensate for Shank2 in *Shank2*-deficient mice, while Shank2 was thought to compensate for Shank3 in another form of *Shank3 *variant homozygous mice, as indicated by the upregulation of Shank3 at the synapse in *Shank2* homozygous mice and *vice versa* [46].

In *Shank3* heterozygotes, there is still one functional copy of *Shank3*, and therefore, a proportional amount of Shank3 protein is present at the synapse [27]. A reduced amount of Shank3 could affect multiple associated proteins [20, 22, 43, 44, 47] and may result in changes in protein composition at the synapse, which may explain functional impairment in *Shank3* heterozygous and homozygous mice [27, 28, 48]. While the Shank family does have highly conserved protein-protein interaction domains, they are not redundant; each Shank protein can have specific mechanisms and proteins it targets [20]. Thus, while replacing one Shank protein with another might prevent the synapse from disassembling, it does not guarantee appropriate functioning. This is well illustrated through the mild behavioral abnormalities in these mice, consistently showing impairments in juvenile reciprocal interactions, novel object recognition, and rotarod performance [27, 28]. The mice also showed some evidence for excess self-grooming, reduced ultrasonic vocalizations, and impaired reversal of water maze learning.

The fact that synapse density was not changed despite the marked LTP deficit may reflect that a portion of the synapses in *Shank3* variant mice may be “silent synapses” that have NMDA but not AMPA receptors, rendering the synapse non-functional [49]. Silent synapses are present in wildtype mice as well, forming during LTP induction and often undergoing remodeling to become functional during LTP maintenance [50, 51]. It is possible that to compensate for the increase in silent synapses in *Shank3*-deficient mice at baseline, some silent synapses undergo remodeling to form perforated synapses, which are thought to have a higher efficacy of synaptic transmission [52, 53]. The presence of increased silent synapses is corroborated by reduced basal synaptic transmission in both *Shank3* heterozygous and homozygous mice [27, 28] and reflects a reduction in AMPA receptors, as they are constitutively active even during basal conditions [54]. In fact, a decrease in GluA1-immunoreactive puncta in CA1 was previously reported in *Shank3* heterozygous mice, indicating a decrease in AMPA receptors [27]. This is substantiated by the significant impairment in the maintenance phase of LTP, which is AMPA receptor-dependent [27].

**Fig. 5:**
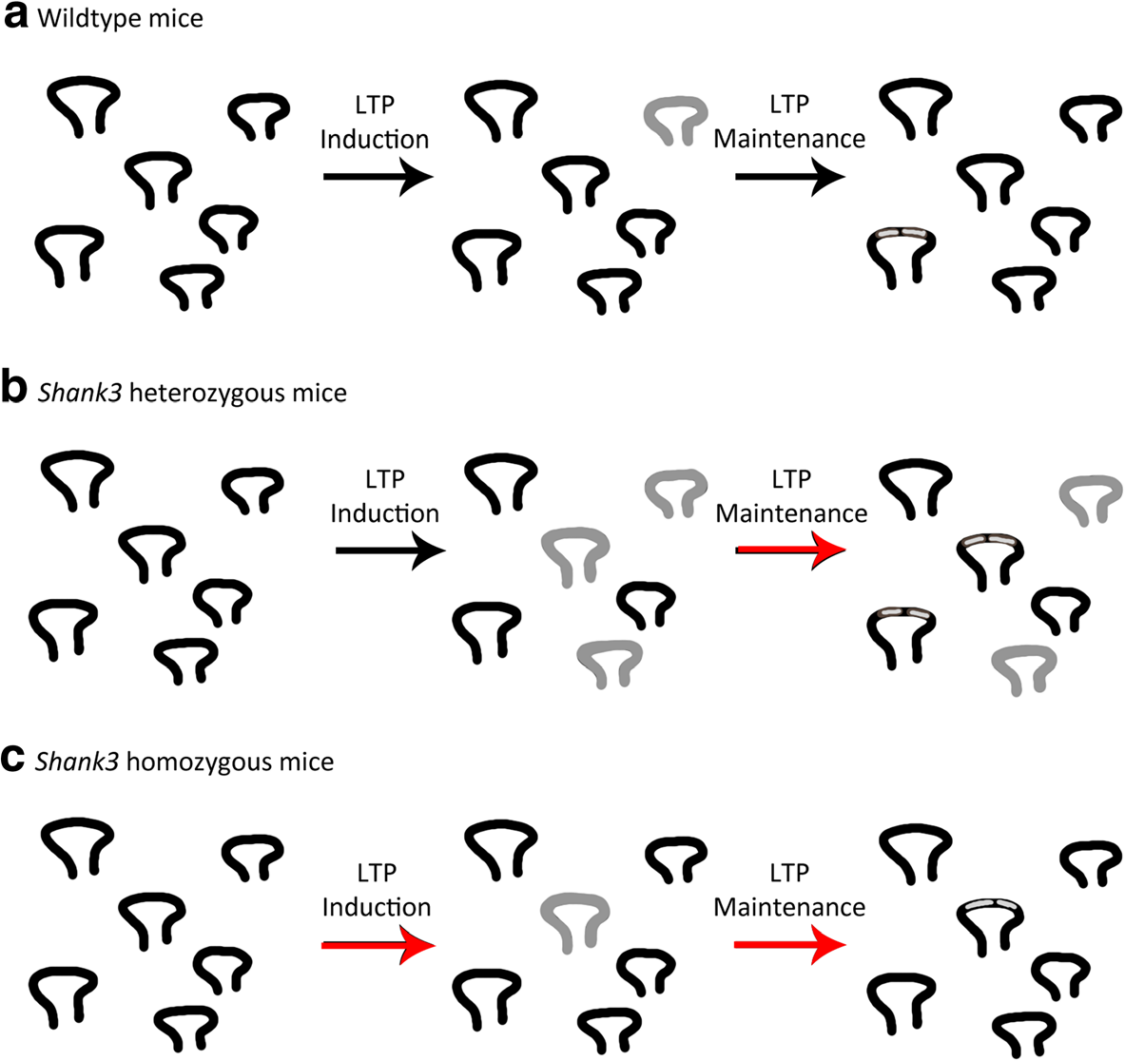
Schematic of suggested mechanism of increased perforated synapse density. **a** Wildtype mice have silent synapses which are remodeled into non-perforated and perforated synapses. **b** *Shank3* heterozygous mice have an increase in perforated synapses, which may not be remodeled into non-perforated synapses due to impaired LTP maintenance. **c** *Shank3* homozygous mice have impairments in LTP induction and maintenance, which may be why the increase in perforated synapses, which is thought to be compensatory, does not occur. *Black synapses* indicate non-perforated synapses; *gray synapses* indicate silent synapses; *black synapses with perforations* indicate perforated synapses. *Red arrows* indicate mechanisms that are impaired

The results of this study indicate that 5-week-old *Shank3* heterozygous mice have a significant increase in perforated synapses. While the overall sample is sizeable for an electron microscopy study, we do realize that an *n* of 5 for each group is small; as such, the caveat that the results may be due to a type I error does arise. In order to resolve this issue, further research with a larger population of mice will be necessary. Nevertheless, the above results suggest the occurrence of a significant increase in perforated spines accompanied by impaired LTP maintenance [27] and of an increase in perforated synapses, which may be providing some, yet insufficient, compensation for an increased amount of silent, non-functional synapses. The remodeling of perforated synapses back into non-perforated synapses occurs during LTP maintenance [50]; however, as LTP maintenance is impaired, this remodeling may not occur, possibly underlying the consistent increase in the amount of perforated synapses. The relative decrease in perforated synapse density to “normal” levels in *Shank3* heterozygous mice at 3 months may be a reflection of a normalization of functional activity with age, as silent synapses can be transiently silent, thereby reducing the need for perforated synapses. Notably, the *Shank3* homozygous mice showed no difference in perforated synapses at 5 weeks (Fig. 2b), despite the substantial impairment in LTP. This may occur because perforated synapses are formed during LTP induction [55], which is significantly impaired in *Shank3* homozygous mice; this impairment may hinder the formation of perforated synapses (see Fig. 5).

While there are no directly correlated studies on synaptic morphology measures in individuals with autism or PMS in the hippocampus, there are several studies that imply alterations at the synaptic level. Neuropathology studies have revealed abnormalities in neuronal numbers [1–6], and imaging studies have revealed a variable degree of altered connectivity localized to specific areas (e.g., [56, 57]). Additionally, work at the protein level has shown abnormalities in receptors in the hippocampus, which may reflect changes at the synapse as well [58, 59]. In accordance with these studies, it will be crucial to use electron microscopy to assess whether there are changes in the synapse at the protein level. Future studies will also expand the age range in these mice, particularly at a younger age as autism is a neurodevelopmental disorder; additionally, expanding the number of mice per group will allow for stronger conclusions regarding the synaptic alterations in these mice.

## Conclusions

This study examined whether synaptic pathology was present in *Shank3* heterozygous and *Shank3* homozygous mice at 5 weeks and 3 months of age. *Shank3* heterozygous mice (5-weeks-old) had a statistically significantly higher density of perforated synapses and no change in other measured parameters (synapse density, PSD length, PSD area, and spine head size); 3-month-old mice had no changes across groups for any measured parameter. It is remarkable that there are so few detectable behavioral and neuropathologic changes in these *Shank3 *variant mice despite the loss of this dominant scaffolding protein. These results highlight the importance of seeking even more subtle alterations, in the form of changes in specific protein levels, at the synapses of these mice. Understanding the subtle effects of this single-locus cause of autism will provide a framework for similar explorations in other genetic causes associated with the disorder, which will allow us to determine whether alterations in these disparate genes lead to similar effects at the most basic functional level, namely, the synapse.

## Abbreviations

AMPA: α-amino-3-hydroxy-5-methyl-4-isoxazole propionic acid
ANOVA: analysis of variance
GluA1: glutamate receptor subunit 1
LTP: long-term potentiation
NMDA: *N*-methyl-D-aspartic acid
PB: phosphate buffer
PBS: phosphate buffer with saline
PMS: Phelan-McDermid syndrome
PSD: postsynaptic density
SHANK3: SH3 and multiple ankyrin repeat domains 3
